# Supplementation with the Prebiotic High-Esterified Pectin Improves Blood Pressure and Cardiovascular Risk Biomarker Profile, Counteracting Metabolic Malprogramming

**DOI:** 10.1021/acs.jafc.2c03143

**Published:** 2022-10-10

**Authors:** Francisco García-Carrizo, Sebastià Galmés, Catalina Picó, Andreu Palou, Ana María Rodríguez

**Affiliations:** † Laboratory of Molecular Biology, Nutrition and Biotechnology (Nutrigenomics, Biomarkers and Risk Evaluation−NuBE), University of the Balearic Islands, 07122 Palma, Spain; § Health Research Institute of the Balearic Islands (IdISBa), 07120 Palma, Spain; ‡ Centro de Investigación Biomédica en Red de Fisiopatología de la Obesidad y Nutrición, Instituto de Salud Carlos III, 28029 Madrid, Spain; ∥ Department of Adipocyte Development and Nutrition (ADE), German Institute of Human Nutrition (DIfE), 14558 Potsdam-Rehbrücke, Germany

**Keywords:** high-esterified pectin, cardiovascular health, microbiota, perinatal malprogramming, prebiotics

## Abstract

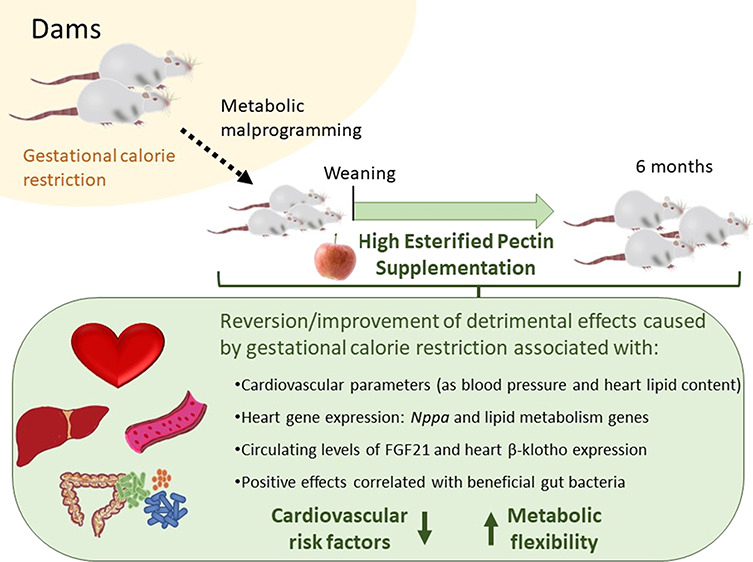

Supplementation with the prebiotic pectin is associated with beneficial health effects. We aimed to characterize the cardioprotective actions of chronic high-esterified pectin (HEP) supplementation (10%) in a model of metabolic malprogramming in rats, prone to obesity and associated disorders: the progeny of mild calorie-restricted dams during the first half of pregnancy. Results show that pectin supplementation reverses metabolic malprogramming associated with gestational undernutrition. In this sense, HEP supplementation improved blood pressure, reduced heart lipid content, and regulated cardiac gene expression of atrial natriuretic peptide and lipid metabolism-related genes. Moreover, it caused an elevation in circulating levels of fibroblast growth factor 21 and a higher expression of its co-receptor β-klotho in the heart. Most effects are correlated with the gut levels of beneficial bacteria promoted by HEP. Therefore, chronic HEP supplementation shows cardioprotective actions, and hence, it is worth considering as a strategy to prevent programmed cardiometabolic alterations.

## Introduction

Cardiovascular disease accounts for almost one-third of all deaths worldwide despite being, in most cases, preventable by addressing behavioral risk factors, such as unhealthy diet, obesity, and sedentarism.^1^ There is growing evidence that the gut microbiota participates in host metabolism, and dysbiosis (an imbalance in the gut microbiota) is associated with cardiovascular disease phenotypes.^2^ Thus, new strategies to modify the gut microbiota by favoring specific species with benefits in reducing cardiovascular disease risk are of interest. In this line, prebiotics have increased as candidates to reduce cardiovascular risk through microbiota modulation.^3^ Among them, pectin has been associated with beneficial effects on metabolic health by reducing calorie intake, modulating chronic inflammation, and reducing post-prandial glycemic response and age-related insulin resistance.^4−7^ Along these lines, we have previously demonstrated that physiological dietary supplementation with the prebiotic high-esterified pectin (HEP) in rats improves adipostatic/adipokine sensitivity and regulates thermogenic capacity, preventing fat gain and deleterious effects associated with metabolic malprogramming and later exposure to an obesogenic diet.^8,9^

Increased body weight and obesity have been consistently associated with increased cardiovascular risk factors, cardiovascular disease,^10^ and gut dysbiosis.^11^ Besides genetic and environmental factors, conditions during the perinatal period are considered causal factors of increased obesity risk in adulthood.^12^ In this regard, there is evidence from epidemiological studies and intervention studies in animal models showing that maternal calorie restriction during gestation may increase the propensity to develop obesity and related chronic diseases in adulthood, with different outcomes depending on the type and severity of restriction, as well as on the gender.^13−16^ In humans, the emblematic study of the Dutch famine of 1944–45 has evidenced the adverse effects of severe gestational undernutrition, showing that men who were exposed to the famine during the first two trimesters of gestation had higher rates of obesity at the age of 19 years.^17^ Notably, people exposed to the Dutch famine during early gestation also had a higher prevalence of coronary heart disease at 50 years of age.^18^ In rats, we have shown that maternal food restriction during gestation, even when it is mild/moderate, during the first 12 days of gestation programs the progeny to altered hypothalamic control of food intake and increased body weight and fat, among other disarrays, mainly in males.^13,14^ Of interest, in the model mentioned above of adverse metabolic programming established in rats, chronic HEP supplementation in the offspring has been shown to prevent excess body weight/adiposity and various adverse metabolic disturbances, which may be related, in part, to an improved profile and sensitivity of the main adipostatic (leptin, insulin, and adiponectin) hormones and the promotion of beneficial bacteria in the gut.^8,9^ However, the effects of this maternal condition on the offspring’s cardiovascular risk and the potential beneficial impact of HEP supplementation have not been assessed.

In this context and taking as a reference a non-infrequent condition in humans, here we aimed to study in rats the effects of mild calorie restriction (20%) during the first 12 days of gestation in the male progeny and their performance under dietary stress (high-sucrose diet) in adulthood. Specifically, we meant to characterize the consequences of mild gestational calorie restriction on cardiovascular risk factors in terms of blood pressure (BP), heart rate and size, lipid content, gene expression, and circulating markers of cardiovascular risk and whether chronic HEP supplementation in the offspring may have a reversal or protective effect against such potential detrimental outcomes.

## Materials and Methods

### Animals and Experimental Design

The animal protocol was evaluated and approved by the Bioethical Committee of the University of Balearic Islands (Res. number 3513). The animals were from a cohort described in previous works.^8,9^ In brief, pregnant Wistar dams were divided into two groups (six rats per group): the control dams’ group, fed ad libitum with standard diet (SD), containing 3.3 kcal/g, with 8% calories from fat and 4% (w/w) of cellulose (Panlab A08, Barcelona, Spain), and the calorie restriction dams’ group, fed with the same diet but with 20% calorie restriction (compared to the total intake of the control group) during 1–12 pregnancy days. The first day after parturition, the number of pups in each litter was adjusted to 10 per mother. From day 21 of life (weaning) to day 135, male offspring were divided into three groups, all fed ad libitum: the control (C) group included the progeny of control dams and were fed with SD, the calorie restriction group was composed of the progeny of calorie-restricted dams and were fed with SD, and the calorie restriction supplemented with pectin (CRP) group was the progeny of calorie-restricted dams and were fed with SD with 10% (w/w) of apple HEP (with 70–75% degree of esterification, molecular weight 30–100 kDa, Sigma-Aldrich Chimie, Lyon, France, ref. 76282). The intake in grams was measured every 2 days, and the feeders were refilled with 100 g of the powder SD hand-operated with pectin (w/w) (90 g of SD + 10 g of pectin). From day 135 until day 180, half of the animals in each group were fed with the same diet but supplemented with 30% sucrose (HS—high-sucrose diet) (final *n* = 6–10 animals/group) (C-HS, CR-HS, and CRP-HS, respectively), and the other half continued with SD (C-SD, CR-SD, and CRP-SD, respectively). The diet was in powder form to facilitate supplementations. In a previous study with the same cohort of animals,^8^ we accurately measured cumulative energy intake for a period of 48 h at 5 months of age. Considering this accurate measure of intake in the adult rats and the percentage of HEP in the diet, we calculated the representative intake of HEP in the adult animals as a reference of daily HEP intake, which is 2.50 ± 0.14 g in the CRP-SD group and 2.39 ± 0.07 g in the CRP-HS group, without statistical differences between the two HEP-supplemented groups (*p* = 0.631, Mann–Whitney *U* test). Finally, all animals were sacrificed at 6 months of age by decapitation for tissue recollection.

### BP and Heart Rate Measurements

BP—systolic (SBP) and diastolic (DBP)—and heart rate of the animals were measured at 5 months of age (*n* = 6 per group) using a non-invasive method based on a rubber inflatable sphygmomanometer with a tail cuff and a photoelectric sensor (NIPREM 546, Cibertec S.A, Madrid, Spain), without anesthesia and after 30-min acclimatization to prevent animal stress hypertension. During this acclimatization time, vasodilation was induced by warming the rat with a red-light bulb. The Niprem V1.8 software was used to determine BP, and the rate values and the mean of at least five measurements per animal were used.

### Peripheral Blood Mononuclear Cell Isolation

Blood samples were collected at 2, 4, and 6 months of age (six animals per group), and peripheral blood mononuclear cells (PBMC) were isolated from total blood by density gradient separation using OptiPrep Density Gradient Medium (Axis-Shield, Dundee, UK) following the manufacturer guides.

### RNA Isolation, Reverse Transcription, and PCR

Total RNA from rats was isolated from the liver, heart, and PBMC at different times using two protocols, depending on the type of tissue or sample. For liver RNA extraction, TriPure Reagent (phenol-based, Roche Diagnostics GmbH, Mannheim, Germany) was used following the manufacturer’s protocol. Total RNA was also extracted from the heart and PBMC (*n* = 6 per group) by E.Z.N.A. Total RNA Kit I (Omega Bio-Tek, Norcross, GA, USA), as the manufacturer’s protocol describes. Isolated RNA was quantified using the spectrophotometer NanoDrop ND-1000 (Nano-Drop Technologies, Wilmington, DE, USA), confirming its integrity by 1% agarose gel electrophoresis. Then, total isolated RNA was reverse transcribed into complementary DNA (cDNA) in an Applied Biosystems 2720 Thermal Cycler (Applied Biosystems, Madrid, Spain) and real-time quantitative polymerase chain reaction (RT-qPCR) with StepOnePlus protocol (Applied Biosystems, Madrid, Spain) was performed to measure mRNA expression levels in the heart, PBMC, and liver, as described previously.^19^ Regarding gene expression in the heart, we studied those genes coding for natriuretic peptides A (*Nppa*), B (*Nppb*), and C (*Nppc*); myostatin (*Mstn*); myocardin (*Myocd*); peroxisome proliferator-activated receptor α (*Ppara*); PPARγ-coactivator 1 α (*Ppargc1a*); 5′-AMP-activated protein kinase (AMPK) catalytic subunit alpha-2 (*Prkaa2*); carnitine palmitoyltransferase 1b (*Cpt1b*); fatty acid synthase (*Fasn*); FGF21 receptor (*Fgfr1*); and co-receptor β-Klotho (*Klb*). Furthermore, the expression mRNA levels of *Nppa* and *Mstn* in PBMC and expression mRNA levels of *Fgf21* in the liver were analyzed. GDP dissociation inhibitor alpha (*Gdi1*) was used as a housekeeping gene for the liver and heart and proteasome subunit alpha type-6 (*Pmsa6*) for PBMC. All primers used were obtained from Sigma-Genosys (Sigma-Aldrich Química S.A., Madrid, Spain), and they are shown in Table S1.

### Determination of Total Lipid and Triacylglyceride Content in the Heart

Total lipid determination was performed by mixing 100–150 mg of heart tissue with 1 mL of hexane/isopropanol (3:2, v/v), following the protocol established by Folch et al.^20^ Tubes with the samples were gassed with nitrogen before being closed to minimize lipid oxidation and then left overnight under orbital agitation at room temperature protected from light. The content of each tube was transferred into a new one, and 0.3 mL of Na_2_SO_4_ (0.47 M) was added and mixed for 5 min, left for 15 min in orbital agitation, and finally centrifuged at 1000 × g for 10 min at 4 °C. The upper phase containing lipids was dissolved in hexane and transferred to a clean, previously weighed glass tube. The hexane extract was then dried with nitrogen gas. Once the tube was dried, the percentage of lipids was determined as the weight difference between tubes with lipid extract and clean tubes, considering the initial amount of tissue present. Triglyceride (TG) content was determined from the lipid extracts dissolved in lipoprotein lipase (LPL) buffer (28.75 mM Pipes, 57.41 mM MgCl_2_·6H_2_O, 0.569 mg/mL bovine serum albumin-fatty acid-free) with sodium dodecyl sulfate 0.1%, as described in the literature^21^). Samples were re-suspended in 3 mL of LPL buffer and were sonicated for 30 s. Tubes were left overnight in an orbital shaker and protected from light at room temperature. The following day, the tubes were coldly sonicated with three pulses of 30 s each. Their TG levels were measured immediately using the Serum Triglyceride Determination Kit (Sigma-Aldrich, Saint Louis, MO, USA), following the manufacturer’s instructions.

### Western Blot for Heart Proteins and Circulating FGF21 Measurement

Western blot was performed to determine the cardiac protein levels of phosphorylated AMPK, the serine/threonine-protein kinase AKT (protein kinase B), adipose triglyceride lipase (ATGL), and CPT1B and cytochrome c oxidase subunit 4 (COX4). A detailed Western blot protocol is described elsewhere.^9^ Briefly, total protein was extracted from the homogenized heart in radioimmunoprecipitation assay (RIPA) lysis buffer, and the protein content was determined by the Bradford method. For SDS-PAGE electrophoresis, 40 μg of total protein per sample was loaded. Electroblotting was carried out with the Trans-Blot Turbo Transfer System (Bio-Rad). For labeling and detection, the specific primary antibodies used are shown in Table S2**.** Antibodies infrared (IR)-dyed 800 or IR-dyed 680LT (LI-COR Biosciences, Lincoln, NE, USA) were used as secondary antibodies. IR was detected by scanning in Odyssey Infrared Imaging System (LI-COR Biosciences, Lincoln, NE, USA), and bands were quantified using the analysis software provided (Odyssey Software V.3.0). ACTB was used as the loading control. Serum FGF21 levels were analyzed under fed conditions at 5 months of age using the ELISA Kit Quantikine Mouse/Rat Immunoassay (R&D Systems, MN, USA).

### Statistical Analysis

Data are expressed as mean ± standard error of mean (SEM). Differences among the C, CR, and CRP groups, under SD or HS diet, were assessed by two-way ANOVA and LSD post hoc analysis. When there was an interaction in the two-way ANOVA, comparisons between groups (splitting by diet) were assessed by one-way ANOVA and LSD post hoc analysis. The statistical assessment of differences between specific groups was carried out by Mann–Whitney *U* test (this non-parametric test was selected as the most suitable since most groups had an n ≤ 10). The significance threshold was set at *p <* 0.05, and *p-*values between 0.05 and 0.10 were considered non-significant tendencies. Analyses were performed with SPSS for Windows (SPSS version 27.0.0, Chicago, IL, USA). For correlation and integrative analysis of principal parameters with gut caecum bacteria relative content and short-chain fatty acid (SCFA) profile, previous data from García-Carrizo et al.^8^ on the profile of bacteria/total bacteria for Firmicutes (*Clostridium coccoides*, *Clostridium leptum*, and *Lactobacillus* spp.), Bacteroidetes (*Bacteroides*/*Prevotella*), Actinobacteria (*Bifidobacterium* spp.), and *Akkermansia muciniphila*; acetate, propionate, and butyrate (SCFAs); and cecum length were included in the analyses of correlation and principal component analysis (PCA). Spearman correlation assessment and PCA were carried out with SPSS v27. Data were normalized to perform PCA. Correlation maps were performed using R Software Package corrplot, following the guidelines of Statistical Tools for High-throughput Data Analysis (STHDA).

## Results

### Pectin Supplementation Reverses Adverse Effects of Maternal CR in BP and Heart Lipid Content of the Progeny

**Figure 1:**
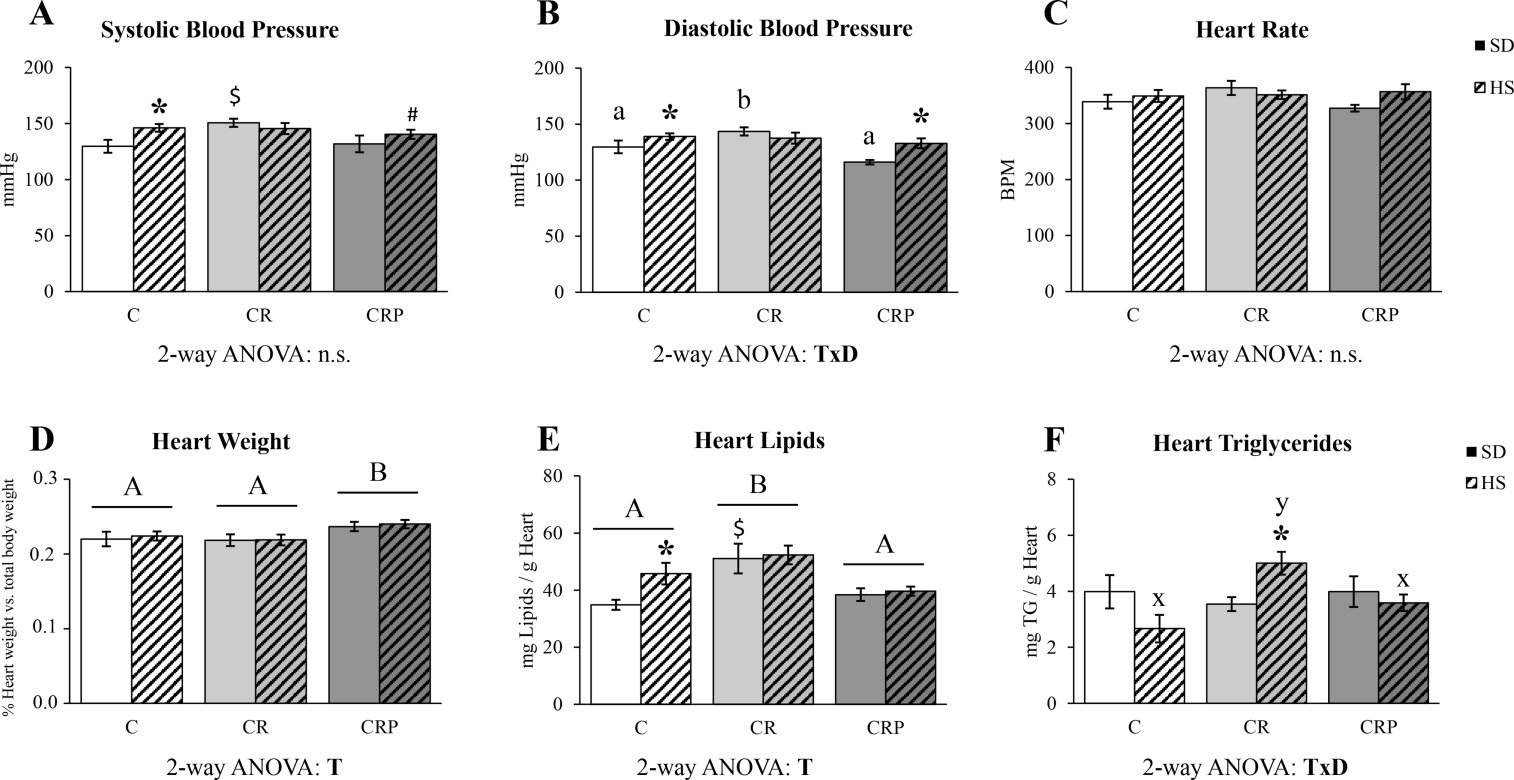
(A) Systolic blood pressure (SBP), (B) diastolic blood pressure (DBP), (C) heart rate in beats per minute (BPM), (D) the percentage of heart weight with respect to total body weight, (E) total heart lipid content, and (F) heart triglyceride (TG) content. SBP, DBP, and heart rate were measured at 5 months of age; heart weight, lipid content, and TG were measured after sacrifice (at 6 months of age). Results are expressed as the mean ± SEM of six to eight animals per group. C, offspring of control dams; CR, offspring of dams subjected to calorie restriction during the first 12 days of pregnancy; CRP, CR rats supplemented with high-esterified pectin between days 21 and 180 of life. SD, standard diet; HS, high-sucrose diet (supplemented between days 135 and 180 of life). Statistics: two-way ANOVA was performed to analyze the effects of Treatment (T) and Diet (D), with LSD post hoc analysis (*A* ≠ *B*). In case of a significant interaction (T×D), one-way ANOVA was performed, with LSD post hoc analysis, splitting individuals with standard diet (*a* ≠ *b*) and HS diet (*x* ≠ *y*). Specific differences between individual groups were assessed by Mann–Whitney *U* test (*p <* 0.05): *HS versus SD, ^$^CR or CRP group versus C group (same diet). n.s., non-significant.

The effects of moderate maternal CR and pectin supplementation, under standard or HS diet, on BP (SBP and DBP), heart rate (at 5 months of age), heart size, and lipid and TG contents (at 6 months of age) in the C, CR, and CRP rats are represented in Figure 1. Concerning BP, C animals under the HS diet showed increased SBP than those fed with SD, and a tendency in this sense was also found in CRP animals (*p* = 0.093). No differences between the animals fed with SD or HS diet were found in the CR group, but CR-SD rats showed increased SBP with respect to C-SD (Figure 1A). For DBP, there was a treatment per diet interaction (T×D): in the C and CRP groups, but not in the CR group, HS diet increased DBP. In addition, CR animals showed increased DBP under the SD, but this effect was reverted in the CRP group (Figure 1B). Hence, the offspring of dams with gestational calorie restriction presented increased BP, not further increased by HS feeding, while HEP supplementation normalized the levels to the control situation. No significant differences were found between groups for heart rate (Figure 1C). However, there was a significant effect of HEP treatment increasing heart size (in terms of % of body weight) (Figure 1D), without effects of HS diet feeding.

Heart total lipid and TG contents in the different experimental groups are shown in Figure 1E,F. Heart lipid content was increased in the CR group with respect to controls. Moreover, the HS diet caused an expected increase in heart lipid content in C animals, an effect lost in the CR animals, which already showed increased lipid content. This effect was reverted by pectin supplementation, which even prevented the HS diet-associated lipid increase (Figure 1E). The amount of the main specific lipid type (TGs) was affected by both treatment and diet with an interactive T×D effect: while under SD, there were no differences between treatments, and under the HS diet, the CR group showed increased heart TG content, an effect reverted, again, by HEP supplementation (Figure 1F).

### Gestational CR Condition and Pectin Supplementation Are Associated with Changes in Gene Expression of Natriuretic Peptides and Myostatin

**Figure 2:**
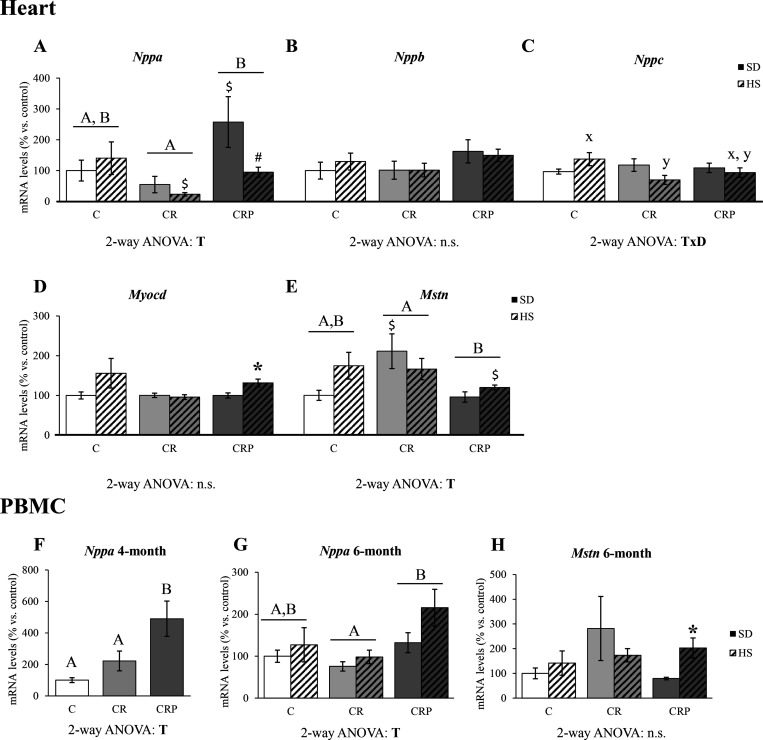
Heart mRNA expression levels of genes coding for natriuretic peptides A (*Nppa*) (A), B (*Nppb*) (B), C (*Nppc*) (C), myocardin (*Myocd*) (D), and myostatin (*Mstn*) (E) at 6 months of age. PBMC expression levels of *Nppa* at 4 (F) and 6 months (G) and of *Mstn* at 6 months of age (H). Results are expressed as a percentage of the mean value of the control group, mean ± SEM of 6 to 10 animals per group. C, offspring of control dams; CR, offspring of dams subjected to calorie restriction during the first 12 days of pregnancy; CRP, CR rats supplemented with high-esterified pectin between days 21 and 180. SD, standard diet; HS, high-sucrose diet (supplemented between days 135 and 180 of life). Statistics: ANOVA and post hoc as explained in Figure 1 legend, *A* ≠ *B*, *x* ≠ *y*, Mann–Whitney *U* test (*p <* 0.05): *HS versus SD, ^$^CR or CRP group versus C group (same diet), ^#^HS versus SD at the *p <* 0.1 level. n.s., non-significant.

The mRNA expression levels of selected genes that may reflect cardiovascular risk status were analyzed in the heart (Figure 2A–E). We focused on heart key genes for cardiomyocyte function and the control of BP, such as those coding for natriuretic peptides,^22^ and for *Mstn* and *Myocd* (related to the control of cardiac muscle growth).^23,24^ Regarding mRNA expression levels of heart natriuretic peptides (*Nppa*, *Nppb*, and *Nppc*, Figure 2A–C), the CR group showed a tendency for decreased levels of *Nppa* mRNA versus controls (*p =* 0.076). Still, HEP supplementation increased the levels of *Nppa* expression above those of the CR group, and the HS diet tended to decrease (*p =* 0.071) its mRNA levels in the CRP animals (Figure 2A). There were no significant changes in *Nppb* mRNA levels in CR animals compared to controls, but there was a tendency for upregulation in the HEP-supplemented rats (*p =* 0.082) (Figure 2B). Regarding the levels of *Nppc* mRNA (Figure 2C), there was an interactive effect of treatment and diet, and when splitting the groups by diet, differences were found between groups under the HS diet, with CR animals showing decreased *Nppc* expression compared to the C group. This effect was partially reverted in the CRP group. Regarding the expressions of *Myocd* and *Mstn*, *Myocd* mRNA levels were significantly increased under the HS diet only in the CRP group (Figure 2D), while *Mstn* expression tended to be increased in the CR animals (the levels were significantly higher in CR animals with respect to C under SD) and was significantly reduced (respect to CR animals) in the CRP group (Figure 2E).

Considering the changes found in the mRNA levels of *Nppa* and *Mstn* in the heart at 6 months of life, in response to gestational CR conditions and/or to HEP supplementation, we considered of interest to analyze their expression levels in PBMC at different ages, to explore their potential interest as biomarkers, and to predict the adverse outcomes associated with CR or the protective role of pectin supplementation. No significant differences between groups were observed regarding the expression levels of *Nppa* (at 2 months) and *Mstn* (at 2 and 4 months) in PBMC (Supplementary Figure 1). However, the treatment did already significantly affect the expression of *Nppa* at 4 months, when all animals were under SD (Figure 2F), with a significant increase in the CRP group compared to the C and CR groups. At 6 months, the PBMC expression of *Nppa* (Figure 2G) was also significantly affected by treatment, with the CRP group having the highest levels significantly different from the CR group. The overall profile of *Nppa* expression in PBMC at 6 months was partially comparable to the expression profile in the heart, particularly regarding the effects of gestational CR and pectin supplementation. Regarding *Mstn* expression, only a significant induction by HS with respect to SD feeding was observed in the CRP group (Figure 2H).

### Diet and Pectin Supplementation Regulate Gene Expression and Protein Activity in the Heart, Potentially Related to the Observed Changes in Lipid Content

**Figure 3:**
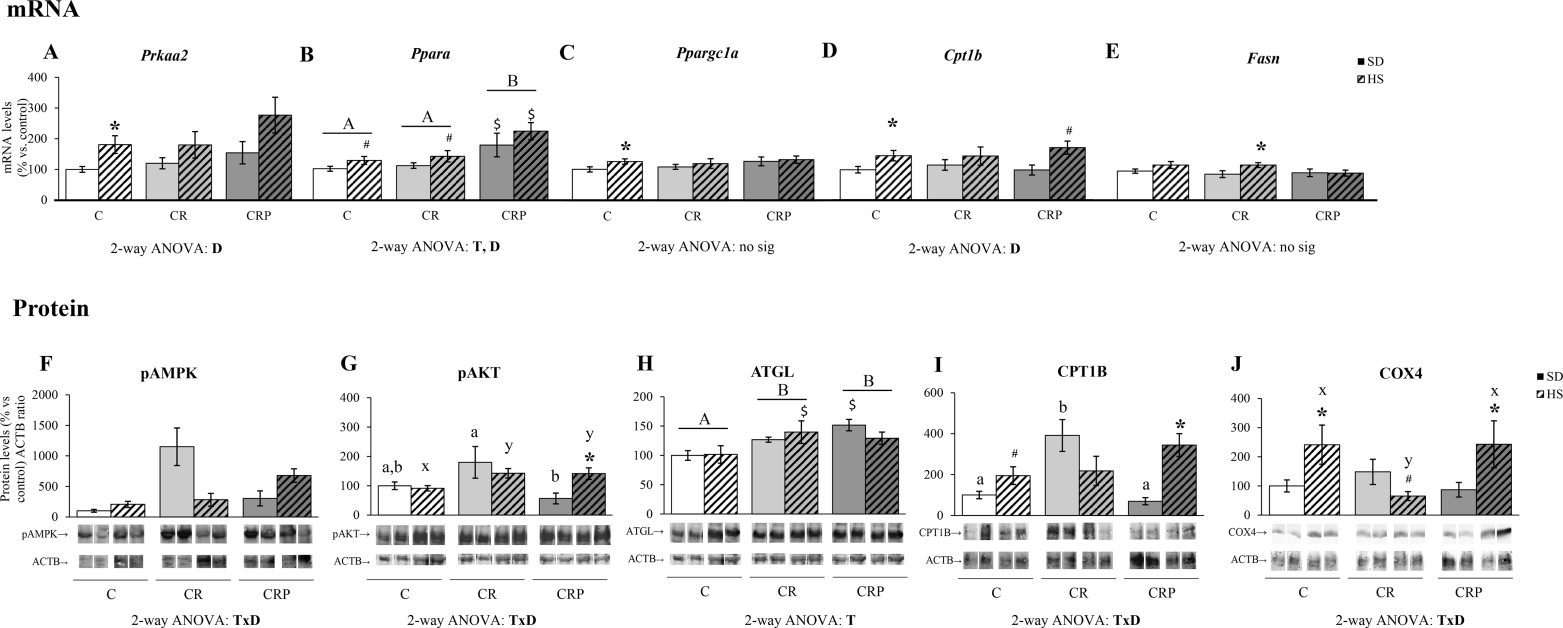
Heart mRNA and protein levels of genes related to lipid oxidation and the control at 6 months of age. (A–E) mRNA levels of the genes Prkaa2 (coding for the AMP-activated protein kinase (AMPK) α subunit), Ppara (for peroxisome proliferator-activated receptor (PPAR) α), Ppargc1a (for PPARγ co-activator 1 α), Cpt1b (for carnitine palmitoyltransferase 1b (CPT1B)), and Fasn (for fatty acid synthase). (F–J) Protein levels of phosphorylated AMPK, phosphorylated AKT, adipose triglyceride lipase (ATGL), CPT1B, and cytochrome c oxidase subunit 4 (COX4). Below the (F)–(J) graphs, representative Western blot images of the corresponding bands are shown: pAMPK 63 kDa, pAKT 60–62 kDa, ATGL 54 kDa, CPT1B 75–85 kDa, COX4 19 kDa, and ACTB 42 kDa. Results are expressed as a percentage of the mean value of the control group, mean ± SEM of 6 to 10 animals per group. C, offspring of control dams; CR, offspring of dams subjected to calorie restriction during the first 12 days of pregnancy; CRP, CR rats supplemented with high-esterified pectin between days 21 and 180. SD, standard diet; HS, high-sucrose diet (supplemented between days 135 and 180 of life). Statistics: ANOVA and post-hoc as explained in Figure 1 legend, *A* ≠ *B*, *a* ≠ *b*, *x* ≠ *y*. Mann–Whitney *U* test (*p <* 0.05): *HS versus SD, $CR or CRP group versus C group (same diet), #HS versus SD at the *p <* 0.1 level. n.s., non-significant.

Linked to changes in total lipid and TG profile content in the heart, the expression (mRNA and protein) of selected genes related to lipid metabolism and/or the activation (phosphorylation) of key signaling molecules were also determined to ascertain potential molecular mechanisms involved. Therefore, *Prkaa2*, *Ppara*, *Pparagc1a*, *Cpt1b*, and *Fasn* mRNA levels were analyzed, and the results are shown in Figure 3A–E. Generally, an effect of HS diet was found upregulating *Prkaa2*, *Ppara*, and *Cpt1b* gene expression. For *Ppargc1a* and *Fasn*, expression was significantly increased only in the C and CR groups, respectively. The induction of these genes under an HS diet was expected, taking into account their involvement in lipid metabolism.^25^ Regarding the effects of treatment, there was a significant increase in *Ppara* expression in the CRP group with respect to both C and CR groups, beyond the HS diet effects.

The activation of AMPKα and AKT was measured by their phosphorylation levels in Thr172 and Ser473, respectively. Interactive T×D effects were observed in both cases, and the groups were split by diet for one-way ANOVA (Figure 3F,G). Under SD, pAMPK levels were significantly increased in the CR animals, an effect reverted by HEP supplementation. In the control and CRP animals, HS diet feeding tended (*p <* 0.1) to increase phosphorylated AMPKα levels, while in CR animals, pAMPKα levels were significantly reduced in response to HS diet; therefore, under the HS diet, the highest pAMK levels were found in the CRP group. In the case of pAKT, CRP animals displayed decreased levels with respect to CR animals under SD, and CR and CRP animals showed higher levels with respect to C under the HS diet. In fact, HS diet feeding increased pAMPK levels in the CRP group. Concerning ATGL protein levels, both CR and CRP groups showed increased levels with respect to control animals (Figure 3H). Regarding CPT1b and COX4 protein levels, there were T×D interactive effects. Therefore, when separating the groups by diet, the one-way ANOVA showed that, under SD, the gestational CR condition triggered the induction of CPT1b, an effect reverted by HEP supplementation (CRP group), but these differences were not observed among animals fed with HS diet (Figure 3I), and there was a significant upregulation of the protein levels in response to HS diet in the CRP animals (which was only a tendency, *p <* 0.1, in the C group). For COX4 levels (Figure 3J), significant differences between treatment groups were manifested in HS diet-fed animals, with CR animals showing lower levels than C, an effect reverted by HEP supplementation. This was mainly because the HS diet significantly upregulated COX4 levels in the C and CRP groups. On the contrary, it tended to reduce its levels in the CR animals, suggesting that the altered response to diet by the gestational CR condition was recovered by HEP supplementation.

### Pectin Supplementation Increases FGF21 Circulating Levels and the Cardiac Expression of Its Specific Co-Receptor β-Klotho

**Figure 4:**
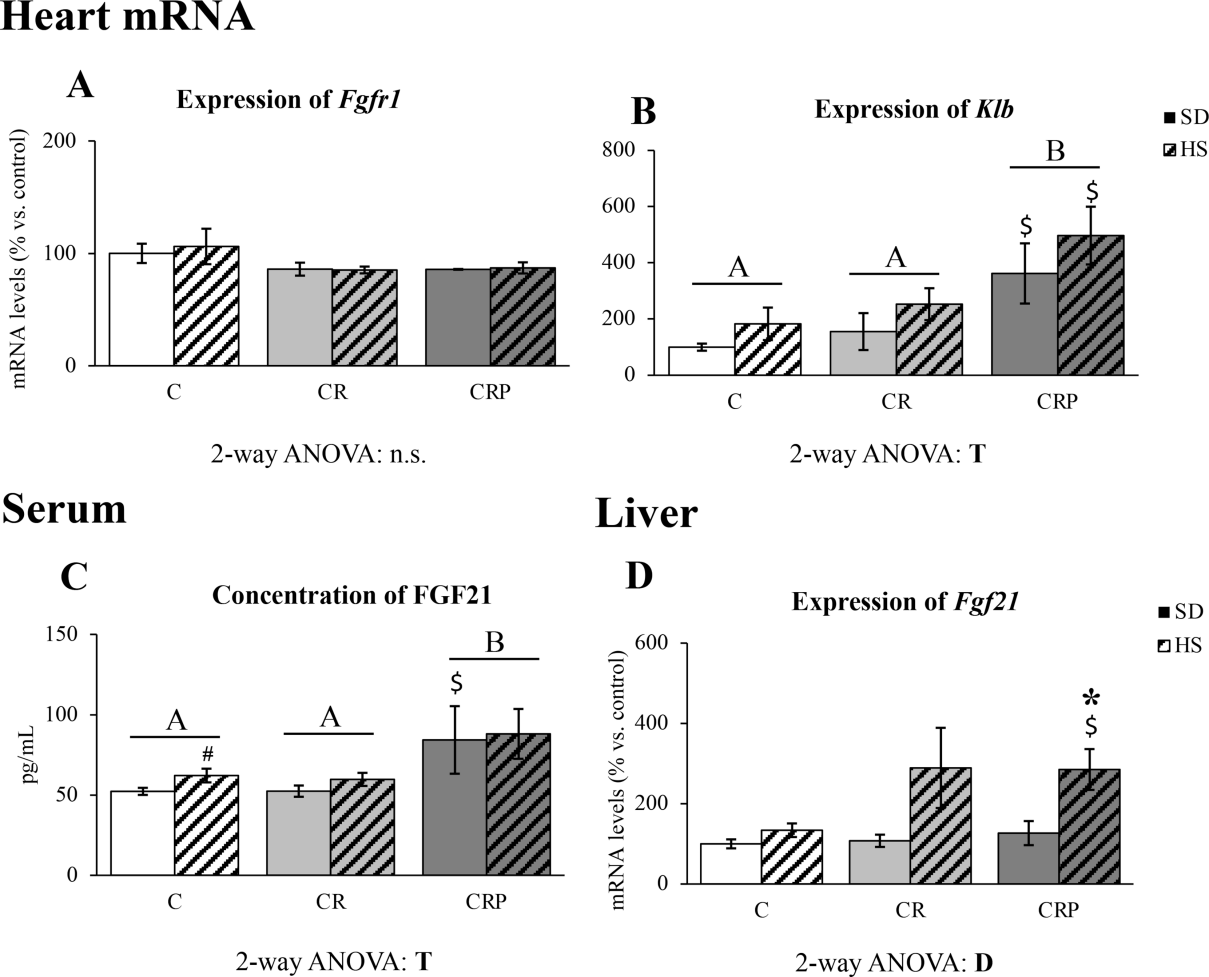
FGF21 and its receptor and co-receptor expression. (A, B) Levels of mRNA of Fgfr1 and Klb in the heart, (C) circulating FGF21 protein, and (D) Fgf21 mRNA in the liver (6 months of age) of the C, CR, and CRP groups under SD or HS diet. Results are expressed as a percentage of the mean value of the control group, mean ± SEM of 6 to 10 animals per group. C, offspring of control dams; CR, offspring of dams subjected to calorie restriction during the first 12 days of pregnancy; CRP, CR rats supplemented with high-esterified pectin between days 21 and 180. SD, standard diet; HS, high-sucrose diet (supplemented between days 135 and 180 of life). Statistics: ANOVA and post-hoc as explained in Figure 1 legend, *A* ≠ *B*. Mann–Whitney *U* test (*p <* 0.05): *HS versus SD, $CR or CRP group versus C group (same diet), #HS versus SD at the *p <* 0.1 level. n.s., non-significant.

The expression (mRNA) levels of *Fgf21* in the liver, the circulating levels of the corresponding protein, and the expression (mRNA) levels of *Fgfr1* and *Klb* (genes for receptor and co-receptor of FGF21, respectively) in the heart are shown in Figure 4. Despite there were neither treatment nor diet effects on heart mRNA levels of *Fgfr1* (Figure 4A), there were clear effects of HEP supplementation on heart *Klb* gene expression (Figure 4B) and on circulating FGF21 levels (Figure 4C), where the CRP group showed higher levels of both parameters compared to the rest of the groups. The HS diet resulted in increased *Ffg21* liver expression, which was significant by Mann–Whitney *U* test only in the CRP group (Figure 4D).

### Correlation and Principal Component Analyses Point out the Relevance of Gut Microbiota Composition as a Mediator of the Pectin Supplementation Impact

Analyses of correlation and PCA were performed to assess potential associations among the most outstanding cardiovascular health-related parameters studied (by the results described above) and the profile of intestinal bacteria, cecum length, and the main SCFAs produced by gut bacteria (acetate, propionate, and butyrate). The data of the levels of gut bacteria relative abundance and SCFA (acetate, propionate, and butyrate) concentration in peripheral blood was published in a previous work.^8^

Briefly, HEP supplementation was associated with increased levels of acetate in peripheral blood in comparison with CR animals. Furthermore, HEP supplementation was also associated with increased caecum abundance of specific beneficial bacteria (including *Bacteroides*/*Prevotella*, *Lactobacillus* spp., and especially *Bifidobacterium* spp.), decreased abundance of potentially detrimental bacteria (*C. coccoides*), and the reversion of gestational CR effects on the levels of *A. muciniphila* (beneficial).^8^

**Figure 5:**
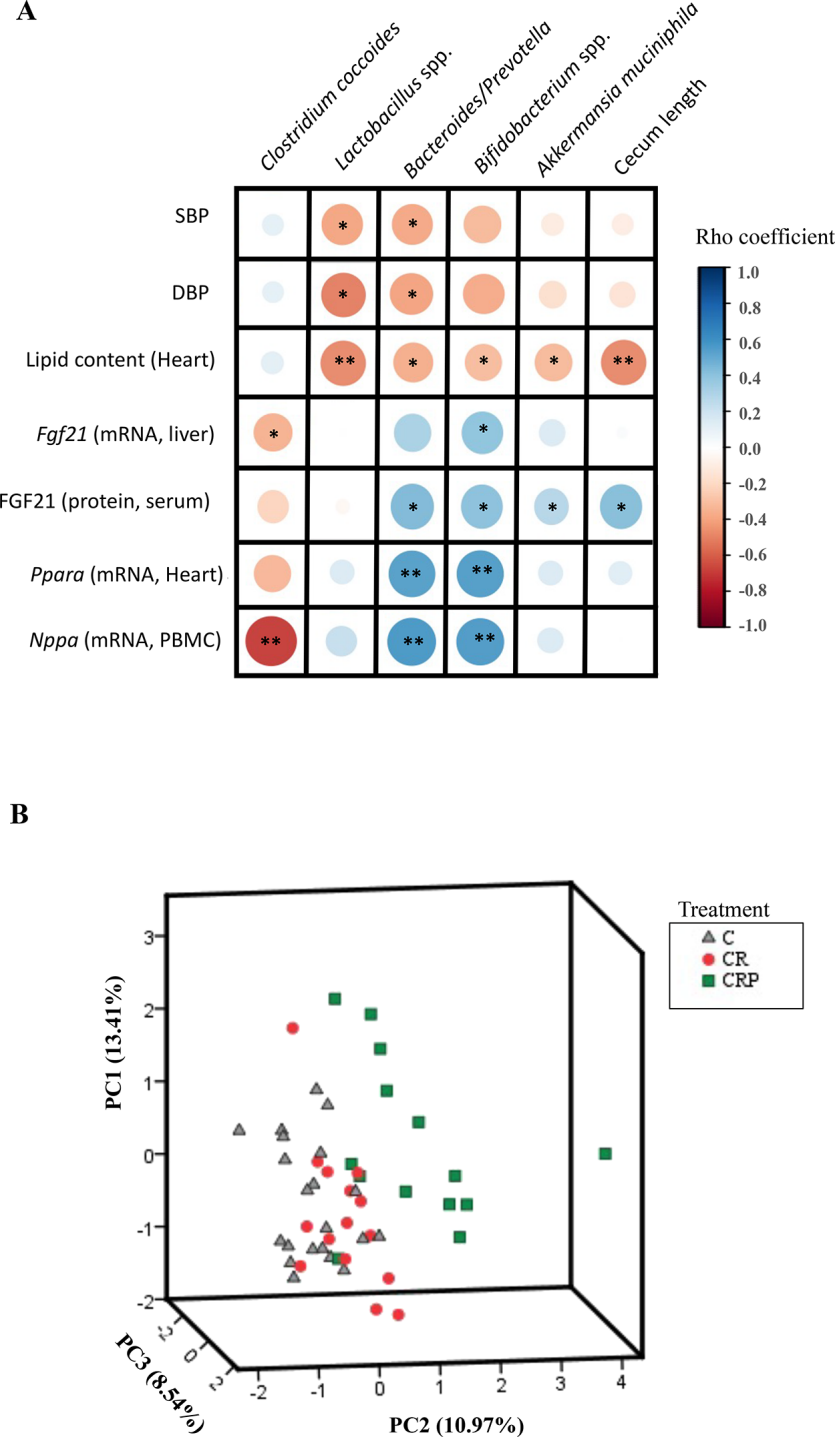
Analyses of correlation analysis and PCA (principal component analysis). (A) Spearman correlation map between the relative abundance of selected health-relevant bacteria (see Materials and Methods section), cecum length, and the cardiovascular health-related parameters studied in the present work. Positive correlations are indicated in blue and negative in red. *Spearman correlation *p-*value <0.05 **p-value <0.01. (B) PCA involves 39 different variables, including the health-related parameters of the present work, the relative abundance of selected health-relevant bacteria, cecum length, and peripheral blood concentration of SCFAs (acetate, propionate, and butyrate). Plots are colored according to the received treatment. The highest positive and negative contributors for PC2 are detailed in the main text (see results section). All data were normalized for PCA performance. Variability explained of PC1, 2, and 3 are indicated next to each axis. Abbreviations: SBP, systolic blood pressure; DBP, diastolic blood pressure; *Fgf21*, fibroblast growth factor 21; *Ppara*, peroxisome proliferator-activated receptor alpha; Nppa, natriuretic peptide A.

Correlation analyses (Figure 5A) revealed a significant inverse association of the relative gut abundance of *Lactobacillus* spp. and *Bacteroides*/*Prevotella* with SBP and DBP and also an inverse association of the relative abundance of *Lactobacillus* spp., *Bacteroides*/*Prevotella Bifidobacterium* spp., *Akkermansia muciniphila*, and cecum length with heart lipid content. The relative levels of *Clostridium coccoides* and *Bifidobacterium* spp. were negatively and positively correlated, respectively, with *Fgf21* liver mRNA levels. In addition, the relative gut abundances of *Bacteroides*/*Prevotella*, *Bifidobacterium* spp., *Akkermansia muciniphila*, and cecum length were positively associated with serum FGF21 levels. *Bacteroides*/*Prevotella* and *Bifidobacterium* spp. relative abundances were positively correlated with mRNA expression levels of heart *Ppara* and PBMC *Nppa*, while *Clostridium coccoides* was inversely associated with mRNA levels of *Nppa* in PBMC.

The PCA elaborated with three main components (PC) was able to explain 32.93% of the observed variability. Although the PCA does not explain a high percentage of variability, the representative plots show a separation of groups, especially in the case of the CRP animals, which more clearly move away from the control and CR animals (Figure 5B). Component 2 (PC2, 10.97% explained variability) allowed the separation of the CRP group from the other groups, with the CRP animals showing the highest values for PC2, while the C and CR groups were set by lower PC2 values. The highest (positive) contributors for PC2 were represented by the relative gut abundance of *Bifidobacterium* spp. (rotate component value: 0.773), *Bacteroides*/*Prevotella* (0.688), serum FGF21 (0.653), *Klb* heart mRNA expression (0.536), *Fgf21* liver mRNA expression (0.454), cecum length (0.448), acetate concentration in peripheral blood (0.413), PBMC *Nppa* mRNA expression (0.403), gut *Lactobacillus* spp. (0.373), and COX4 protein levels in the heart (0.341). Otherwise, the most negative contribution for PCA2 was shaped by the gut relative abundance of *Clostridium leptum* (−0.653) and *C. coccoides* (−0.535), *Fas* (−0.462) and *Ffgr1* (−0.286) mRNA heart expression, heart lipid content (−0.234), DBP (−0.223), SBP (−0.187), *Myocd* (−0.148) and *Nppc* heart mRNA expression (−0.132), and TG content in the heart (−0.116).

All in all, the results derived from the correlation analysis and PCA suggest that changes in cardiovascular health-related parameters associated with HEP supplementation could be intimately related to positive changes in gut bacterial composition.

## Discussion

As shown in previous works,^7−9^ chronic HEP supplementation can ameliorate metabolic disturbances produced by perinatal malprogramming, associated with specific gut microbiota selection, modulating the beneficial/detrimental gut bacterial species balance. These changes have implications in leptin and insulin sensitivity, energy metabolism, and thermogenic capacity.^8,9^ Along these lines, we aimed to study the potential beneficial effects of HEP supplementation in cardiovascular protection, studying the progeny of calorie-restricted (20%) dams during the first half of pregnancy (CR animals), using the same animal cohort as in previous works.^8,9^ The results suggest that HEP supplementation can reduce or counteract cardiovascular risk factors associated with metabolic malprogramming. The potential cardiovascular protective effects of pectin supplementation may be achieved in different ways, as discussed below.

We show here that mild gestational CR caused a significant increase in BP (both SBP and DBP) in the adult offspring, evidenced under SD, which was accompanied by a significant increase in total heart lipid content and a misbalance in TG management under an HS diet, factors that can be associated with cardiac dysfunction and increased cardiovascular risk.^26,27^ However, chronic HEP supplementation clearly counteracted the impairments mentioned above and may even show a further protective role against the damages of the obesogenic HS diet. The reversion of increased BP by HEP supplementation was more evident for DBP since the CRP group under SD displayed significantly lower values than the CR group. It can be noted that the repercussion of SBP and DBP in cardiovascular disease development may be different; e.g., variability in DBP has been recently suggested as a more important predictor of cardiovascular adverse events than SBP in certain patients (with stroke), and DBP and isolated diastolic hypertension seem to be more related to the drive of coronary risk in younger subjects.^27,28^ Moreover, the pectin-supplemented animals showed a higher percentage of heart weight than both C and CR animals. Although cardiac hypertrophy is usually considered a risk factor, the surrounding observed physiological conditions suggest that such increase in the relative heart weight might be associated with a favorable cardiovascular profile, as may happen, for instance, in trained athletes.^29^

To better characterize the cardio benefits of pectin supplementation at a molecular level, the expression levels of genes encoding for natriuretic peptides or involved in heart size regulation were analyzed. On the one hand, natriuretic peptides play a central role in regulating BP and cardiovascular homeostasis, and dysregulation of these peptides could play a major role in disorders such as hypertension, heart failure, and obesity.^22^ Here, pectin supplementation triggered significant increases in the expression of *Nppa* (which codes for natriuretic peptide A—ANP) compared with the CR group, reverting the tendency to downregulation caused by the CR condition, which might be related to the lower BP levels in the HEP-supplemented (CRP) animals compared to CR animals, considering the central role of ANP lowering BP.^30^ Moreover, it has also been described that the cardiac ventricular expression of ANP is decreased in genetically obese or high-fat diet-fed mice, which also show increased cardiac TG content; the same authors reported that TG accumulation in cultured atrial myocytes is accompanied by downregulation of ANP mRNA.^31^ Therefore, our results regarding the lipid content profile in heart and *Nppa* expression are in line with such reports. The induction (respect to CR animals) of *Nppa* expression in the pectin-supplemented animals may be considered as another of the beneficial effects reverting gestational CR malprogramming. However, it was lost when the animals were exposed to an HS diet. On the other hand, *Myocd*- and *Mstn*-encoded proteins (myocardin and myostatin) play a significant role in cardiac morphogenesis, contractility, and heart energy homeostasis.^23,24^ Myocardin is essential for heart development and cardiomyocyte differentiation, but it is also involved in cardiomyocyte hypertrophy.^23^ During cardiac hypertrophy, a phenomenon of “fetal gene activation” is given, suggested as a protective physiological response against stress. The transcriptional co-activator myocardin has been proposed as fundamental in inducing the fetal gene program and cardiac hypertrophy.^32^ Our results show that only CRP animals under the HS stimulus were able to significantly increase the levels of *Myocd* expression, suggesting that pectin supplementation might allow an improved molecular response to metabolic stress. Myostatin is a growth/differentiation factor that is a negative regulator of skeletal muscle mass. Its increased expression in the heart is involved in the pathogenesis of myopathy related to heart failure.^24^ Here, the increase in *Mstn* mRNA levels in the heart due to metabolic programming effects of gestational CR condition (specially observed under SD) was reversed by pectin supplementation. Although the pectin-supplemented animals showed lower *Mstn* mRNA levels with respect to CR animals, the differential profile of response to the experimental conditions with respect to relative heart weight points that it would not be a key factor explaining the increased percentage of heart weight of the CRP animals. Altogether, the beneficial effects of pectin modulating BP may be related, at least in part, to the modulation of specific genes in the heart and especially to the induction of *Nppa* expression.

Due to the observed changes in both *Nppa* and *Mstn* mRNA levels in the heart, we considered of interest to study their expression at different ages in PBMC, trying to search for new biomarkers able to predict later disease outcomes in an accessible biological material (blood). Only *Nppa* mRNA levels in PBMC showed significant changes at a relatively early age (4 months of age), partially related to the later changes observed in cardiac expression and BP at 6 months. Therefore, considering both our results and the importance of *Nppa* expression in the heart regarding BP regulation and prevention of cardiometabolic diseases,^22^*Nppa* expression in PBMC may be of interest as a possible health biomarker that deserves more studies to confirm its suitability and utility.

Excess of lipid accumulation in heart cells is associated with lipotoxicity and the development of cardiac dysfunction and cardiomyopathies.^33^ Due to the slight capacity of the heart to store substrates, the control of energy uptake flux from food, together with energy production and demand, is tightly controlled by mechanisms that induce genes encoding molecular regulators of energy metabolism.^34^ In this sense, the increased total lipid and TG (under HS diet) accumulation in the heart of the CR animals suggests an impairment in heart lipid metabolism regulation due to fetal malprogramming. The dysregulation of lipid content observed was accompanied by a series of changes in the expression of key genes and in the activity of master signaling proteins. Still, there were also interesting changes associated with pectin supplementation. In this way, the results show that the HS diet upregulated the expression of *Prkaa2*, *Ppargc1a*, and *Cpt1b* significantly in the C animals while not in the other (CR, CRP) groups. On the contrary, *Fasn* mRNA showed a significant upregulation in response to HS diet only in the CR group, prevented in pectin-supplemented animals (CRP group). Considering the lipogenic role of *Fasn*-encoded protein (fatty acid synthase), such response pattern may be partially responsible for the lower heart lipid content in CRP animals with respect to CR, especially in those fed with HS diet. In addition, the increase in *Ppara* (involved in the transcriptional regulation of fatty acid oxidation^35^) mRNA levels driven by HEP supplementation may also make a significant contribution to avoiding excess lipid accumulation in the CRP animals. Accordingly, this increase may be a factor related to the changes observed in the levels of key proteins involved in lipid catabolism and particularly associated with their capacity to respond to the HS diet. ATGL protein levels were increased in CR animals compared to controls and even more increased with pectin supplementation (in this case, when only considering the animals not exposed to HS diet). Given the role of ATGL as the first enzyme in the process of TG lipolysis,^36^ this could be understood as a physiological, metabolic adaptation (in CR animals) to increase lipid catabolism and therefore avoid excess lipid accumulation in the heart, an event slightly potentiated by pectin supplementation. The same argument could apply to the increase in CPT1B protein levels in CR animals since CPT1B is the main enzyme regulating the entry of long-chain fatty acids to the mitochondria for their oxidation.^25^ However, in this case, the physiological capacity to increase lipid oxidation in response to metabolic stress (HS diet) seemed impaired in CR animals, which did not further increase CPT1B levels. This response was recovered/potentiated in the CRP animals, which showed similar CPT1B levels to control animals under SD but which significantly increased in response to the obesogenic HS diet. A similar situation was given for the expression levels of the mitochondrial respiratory chain protein COX4 (used here as an indicator of oxidative capacity) since they tended to be downregulated in response to the HS stimulus in CR animals. In contrast, the opposite was observed in control animals—a response recovered in animals with pectin supplementation (CRP animals). Altogether, these results suggest that lipid oxidation control (and the capacity to respond to metabolic stress) is altered by the malprogramming caused by the gestational CR condition. Still, pectin supplementation allows the recovery of the metabolic flexibility in the heart and may even increase it.

The activation (phosphorylation) state of the master metabolic regulator kinases AMPK and AKT also point to cardiometabolic protective effects of HEP supplementation. Phosphorylation of AMPKα (the catalytic subunit) was altered in CR animals. The control pAMPKα levels and response to HS diet were recovered in the pectin (CRP)-supplemented animals. In this sense, the activation of AMPK in the heart might be suggested as a physiological response to the metabolic stress imposed by the HS diet since activated AMPK in the heart can favor processes such as glucose transport, glycolysis, and fatty acid oxidation.^37^ Our results suggest an impaired response to HS diet in CR animals but recovered in pectin-supplemented animals. In the case of pAKT, it showed increased levels in CR animals with respect to controls, but only in the HS groups, while the basal (under SD) levels were lower in the CRP animals with respect to CR but significantly increased in response to HS diet, also suggesting a possible improved metabolic flexibility in the HEP-supplemented group. FGF21 has been suggested to have multiple physiological functions, including protecting from cardiomyopathy by diminishing cardiac hypertrophy and oxidative stress in the heart.^38^ FGF21 is mainly produced by the liver and is released into the bloodstream.^39^ We report here that pectin supplementation increased *Fgf21* expression in the liver in response to the HS diet but only significantly in the pectin-supplemented animals. Moreover, a significant increase in FGF21 protein levels released into the bloodstream was observed in the CRP animals with respect to the C and CR groups. An effective response of FGF21 in heart tissue is determined by the presence of specific receptors, especially when they form a complex with the β-Klotho co-receptor, which confers a specific response capacity to FGF21 action.^39^ In this sense, pectin supplementation also stimulated the upregulation of the expression of *Klb* in the heart, without changes in *Fgfr1* mRNA levels. Moreover, FGF21 cardio-protection is linked to the appropriate function of AMPK and AKT activation in the heart.^40^ As shown above, CR animals presented a dysregulation of AKT and AMPK phosphorylation, which was corrected or even improved with pectin supplementation. We suggest that, in our model, the increase in FGF21 levels in the blood, accompanied by the increase in *Klb* expression, may be partly responsible for the described protective effects of pectin supplementation, counteracting gestational CR-programmed cardiovascular risk.

Finally, the correlation analysis and PCA suggest that the HEP-supplemented group of animals tends to separate from the other two groups (C and CR) in its metabolic response and how the main beneficial outcomes of pectin supplementation described here in cardiovascular health-related parameters were positively and negatively correlated with the relative abundance of beneficial (*Lactobacillus* spp., *Bacteroides*/*Prevotella*, *Bifidobacterium* spp., and *Akkermansia muciniphila*) and detrimental (*Clostridium coccoides*) bacteria, respectively (e.g., BP levels were inversely correlated with the relative gut abundance of *Lactobacillus* spp. and *Bacteroides*/*Prevotella*; i.e., lower BP, a health positive effect, was associated with higher levels of these beneficial bacteria). Therefore, it is suggested that the significant positive modulation of the gut microbiota caused by HEP supplementation could play a relevant role in the beneficial cardiovascular effects described in our model.

**Figure 6:**
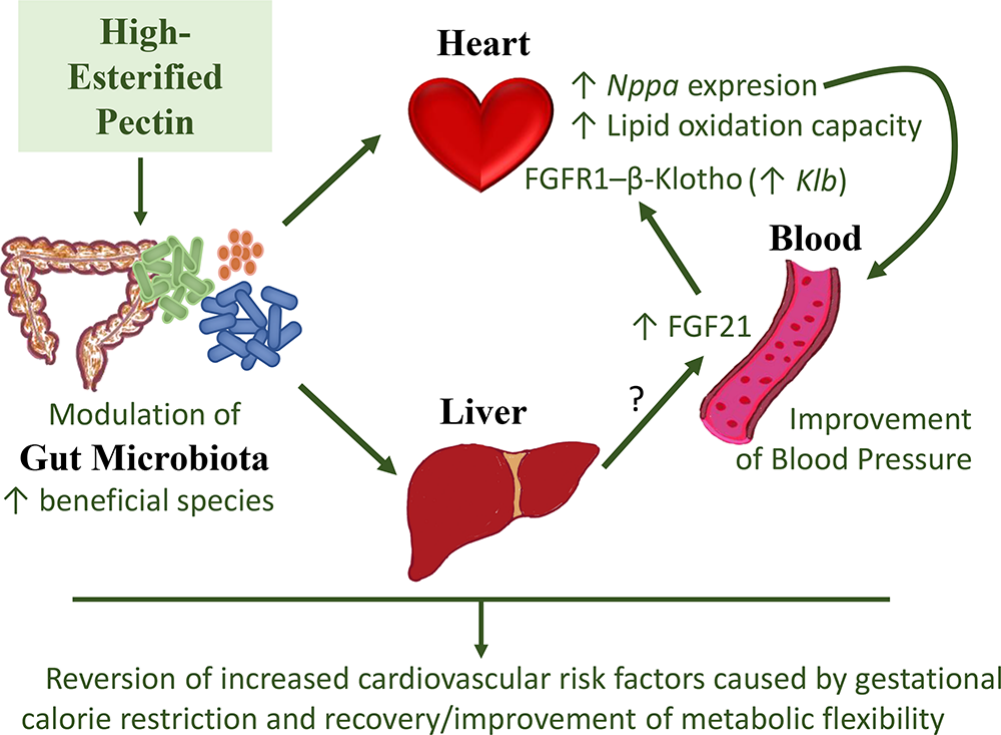
Summary of suggested mechanisms involved in the cardiovascular improvement in gestational calorie-restricted animals, associated with high-esterified pectin (HEP) chronic supplementation. HEP supplementation promotes the modulation of the gut microbiota by favoring the increase in the relative abundance of beneficial species. The changes may indirectly impact critical organs, such as the heart and the liver, modulating gene expression and increasing FGF21 circulating levels. Although the liver is the main productor of FGF21, from our results we cannot distinguish whether the elevated blood levels are caused by increased hepatic secretion or by other tissue/s. FGF21, via its specific receptor FGFR1 and co-receptor β-Klotho, might be partly responsible for the improvements observed in the heart, such as the increase in lipid oxidation capacity and in *Nppa* expression, which in turn would improve blood pressure. Overall, HEP supplementation reverses the increased cardiovascular risk factors caused by gestational calorie restriction and allows the recovery, or even improvement, of metabolic flexibility.

In summary, the present study provides evidence that mild CR during the first half of pregnancy increases cardiovascular risk in the progeny in terms of BP, heart lipid content, and gene expression biomarkers related to cardiac function. However, HEP supplementation can restore and even improve the basal control conditions, thus reducing the cardiovascular risk. The cardiac health improvement driven by pectin supplementation may be explained, at least in part, by modulation of the expression of natriuretic peptides and lipid oxidative capacity in the heart, which in turn may be partially explained by an increase in liver FGF21 production and its possible effects on the heart through its specific co-receptor β-Klotho. We also propose the role of specific microbiota selection by pectin supplementation as an underlying mechanism of the cardiovascular benefits observed (Figure 6). All in all, the present work raises the possibility that HEP may become an interesting bioactive compound in the diet, able to provide protection against the increased cardiovascular risk associated with adverse metabolic programming. These results also support the interest in promoting the intake of fruits rich in pectins and in examining the possible interaction of these compounds with other bioactives present in fruits to make more targeted recommendations to prevent cardiovascular diseases, which represent one of the main causes of morbidity and mortality in humans.

## Supporting Information Available

List of primers used for qPCR determinations; list of primary antibodies used for Western blot analysis; list of abbreviations; PBMC expression levels of *Nppa* (at 2 months of age) and of *Mstn* (at 2 and 4 months of age) (PDF)
