# Supplementary material for: Supplementation with the Prebiotic High-Esterified Pectin Improves Blood Pressure and Cardiovascular Risk Biomarker Profile, Counteracting Metabolic Malprogramming
Source: J Agric Food Chem. 2022 Oct 10;70(41):13200–11. doi: 10.1021/acs.jafc.2c03143 (PMC9585587; doi:10.1021/acs.jafc.2c03143)

Supplementary material

Supplementary Table 1. List of primers used for *q*PCR determinations.

Gene	Gene name	Forward (5' to 3')	Reverse (5' to 3')	Amplicon size (pb)
Cpt1b	Carnitine palmitoyltransferase 1b	GCTCGCACATTACAAGGACAT	TGGACACCACATAGAGGCAG	250
Fasn	Fatty acid synthase	CGGCGAGTCTATGCCACTAT	ACACAGGGACCGAGTAATGC	222
Fgfr1	Fibroblast growth factor receptor 1	TCCGCAGACAGGTAACAGTG	AGCTAGCATGGGGGTTCC	107
Gdi1	GDP dissociation inhibitor 1	CCGCACAAGGCAAATACATC	GACTCTCTGAACCGTCATCAA	159
Klb	Klotho beta	CCCTCGAACACCGTGGTAAA	AACCAGCCGTTCTCCGAAAT	119
Mstn	Myostatin	AGCTGCGCCTGGAAACAGCTC	TTCCCGGAGTGGAGGCGCTC	80
Myocd	Myocardin	CCGTGAAAGAGGCTATAAAAGG	TTCCCGGAGTGGAGGCGCTC	76
Nppa	Natriuretic peptide A	CACAGATCTGATGGATTTCAAG A	CCTCATCTTCTACCGGCATC	68
Nppb	Natriuretic peptide B	GTCAGTCGCTTGGGCTGT	CCAGAGCTGGGGAAAGAAG	105
Nppc	Natriuretic peptide C	AGCGGTCTGGGATGTTAGTG	CCAAGGATGACCTCAGTGC	75
Pmsa6	Proteasome subunit alpha 6	TGGCTATGAGATTCCTGTGG	CTGTCTGCTTCACTCCTGCT	206
Ppara	Peroxisome proliferator activate response alpha	TGTCGAATATGTGGGGACAA	AAACGGATTGCATTGTGTGA	215
Ppargc1a	PPAR gamma, coactivator 1 alpha	AGGAGGGTCATCGTTTGTGG	GGAGGCAGAAGAGCCGTC	256
Prkaa2	Protein kinase, 5'- AMP-activated, alpha 2	CCAAGTGATCAGCACTCCAA	CAACACGTTCTCTGGCTTCA	199

Supplementary Table 2. List of primary antibodies used for Western Blot analysis.

Protein	Antibody for protein complete name	Company	Reference
ACTB	Anti- Beta-Actin	Cell Signaling Technology	3700
ATGL	Anti- Adipose Triglyceride Lipase	Cayman chemical	10006409
COXIV	Anti- Cytochrome c oxidase subunit 4	Cell Signaling Technology	4844
CPT1	Anti- Carnitine Palmitoyl Transferase 1	Santa Cruz Biotechnology	sc-98834
pAKT	Anti- v-AKT murine Thymoma Viral Oncogene homolog 1 phosphorylated at serine 473	Cell Signaling Technology	4051
pAMPK	Anti- AMP-Activated Protein kinase phosphorylated at threonine 172	Cell Signaling Technology	2535

List of Abbreviations

ACTB	β-actin	Klb	β-Klotho
AKT	Protein kinase B	mRNA	Messenger RNA
AMPK	5'-AMP-activated protein kinase	Mstn	Myostatin
ANOVA	Analysis of variance	Myocd	Myocardin
ATGL	Adipose triglyceride lipase	Nppa	Natriuretic peptide A
BP	Blood pressure	Nppb	Natriuretic peptide B
COX4	Cytochrome c oxidase subunit 4	Nppc	Natriuretic peptide C
Cpt1b	Carnitine palmitoyltransferase 1b	PBMC	Peripheral Blood Mononuclear Cell
CR	Calorie restriction (group)	PCA	Principal Components Analysis
C	Control (group)	PCR	Polymerase chain reaction
CRP	Calorie restriction supplemented with pectin (group)	Pmsa6	Proteasome subunit alpha type-6
DBP	Diastolic blood pressure	Ppara	Peroxisome proliferator-activated receptor α
DMS	Minimum Significant Differences	Ppargc1a	PPARγ-coactivator 1 α
DNA	Deoxyribonucleic acid	Prkaa2	AMPK catalytic subunit alpha-2
Fasn	Fatty acid synthase	RNA	Ribonucleic acid
FGF21	Fibroblast growth factor 21	SBP	Systolic blood pressure
Fgfr1	FGF21 receptor	SCFA	Short-chain fatty acids
Gdi1	GDP dissociation inhibitor alpha	SD	Standard diet
HEP	High esterified pectin	SEM	Standard error of mean
HS	High-sucrose diet	TG	Triglycerides

Supplementary Figure 1

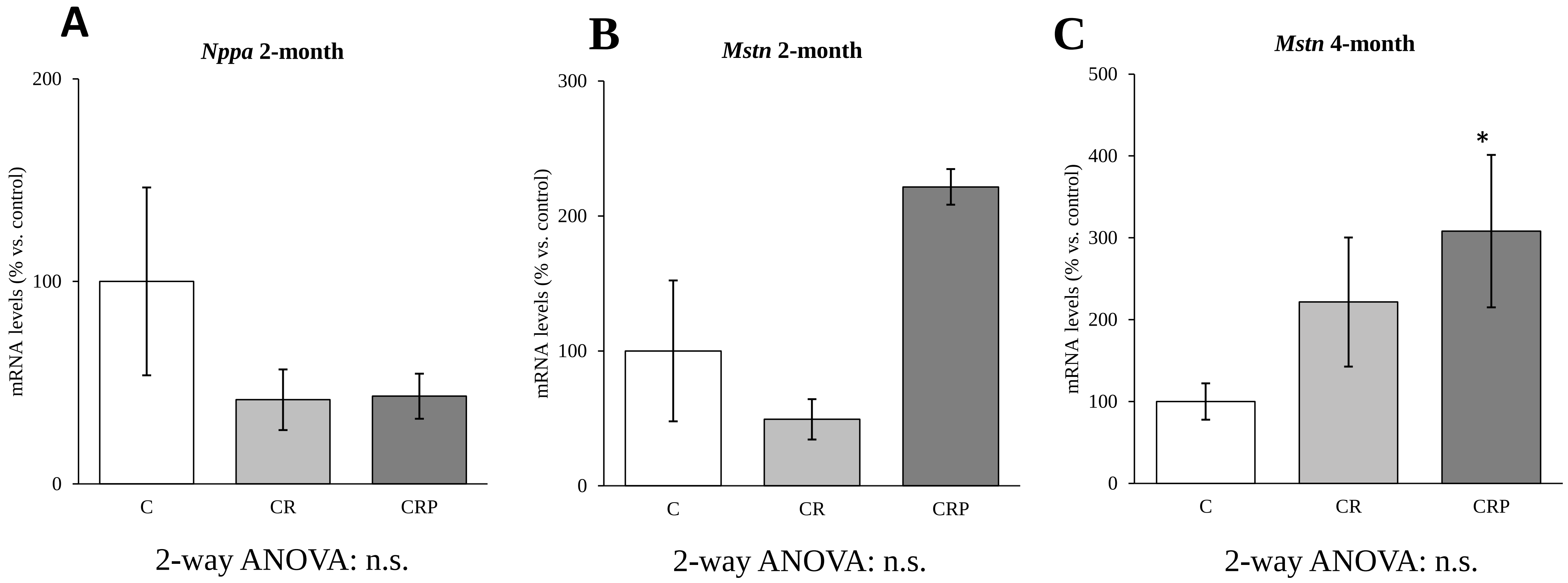

Supplement: Supplementary file 1 — jf2c03143_si_001.pdf [file jf2c03143_si_001.pdf]
